# Physical Implementation of Reservoir Computing through Electrochemical Reaction

**DOI:** 10.1002/advs.202104076

**Published:** 2021-12-29

**Authors:** Shaohua Kan, Kohei Nakajima, Tetsuya Asai, Megumi Akai‐Kasaya

**Affiliations:** ^1^ Graduate School of Information Science and Technology Hokkaido University Kita 14, Nishi 9, Kita‐ku Sapporo Hokkaido 060‐0814 Japan; ^2^ Graduate School of Information Science and Technology The University of Tokyo 7‐3‐1 Hongo, Bunkyo‐ku Tokyo 113‐8656 Japan; ^3^ AI Center The University of Tokyo 7‐3‐1, Hongo, Bunkyo‐ku Tokyo 113‐8656 Japan; ^4^ Graduate School of Science Osaka University 1‐1 Machikaneyama Toyonaka Osaka 560‐0043 Japan

**Keywords:** electrochemical reactions, ionic currents, multiway data acquisition systems, reservoir computing

## Abstract

Nonlinear dynamical systems serving reservoir computing enrich the physical implementation of computing systems. A method for building physical reservoirs from electrochemical reactions is provided, and the potential of chemical dynamics as computing resources is shown. The essence of signal processing in such systems includes various degrees of ionic currents which pass through the solution as well as the electrochemical current detected based on a multiway data acquisition system to achieve switchable and parallel testing. The results show that they have respective advantages in periodic signals and temporal dynamic signals. Polyoxometalate molecule in the solution increases the diversity of the response current and thus improves their abilities to predict periodic signals. Conversely, distilled water exhibits great computing power in solving a second‐order nonlinear problem. It is expected that these results will lead to further exploration of ionic conductance as a nonlinear dynamical system and provide more support for novel devices as computing resources.

## Graphical abstract

Information processing ability of physical reservoirs utilizing electrochemical reactions in faradic current is investigated. Strong redox reactions of the acidic molecule exhibit advantage for the interpretation of periodic signals. Distilled water exhibits great computing ability for solving higher‐order nonlinear problems. The simplicity of such a system will blaze a trail for developing computing systems based on electrochemical ions reactions.

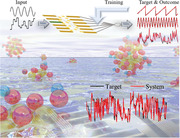

## 1 Introduction

**Figure 1:**
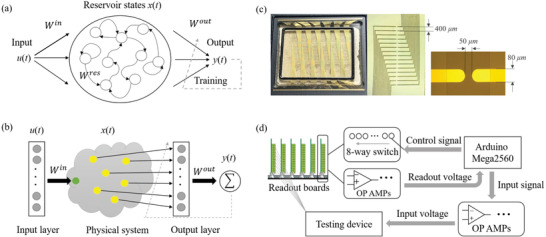
Illustration of physical reservoir computing (RC) and construction of molecular‐based reservoir. a) Structure of traditional reservoir computing. b) Concept of our physical RC system. c) Testing device of planar electrodes. d) Measurement process chart of proposed solution reservoir.

It is becoming increasingly difficult to satisfy the requirements of high efficiency for computing power and storage capacity while developing hardware systems in artificial neural networks. Reservoir computing (RC) originates from recurrent neural networks (RNNs) with input, hidden, and output layers, and has attracted considerable attention owing to its unique advantages.^[^ ^1^ ^]^ First, the output layer of RC is the only part that needs to be tuned toward the target signal, making the learning process faster and simpler while the computing power is no less than that of conventional RNNs.^[^ ^2^ ^]^ Another attractive aspect of RC is that the hidden layer is replaced by a massive number of nonlinear nodes coupled with one another (referred to as reservoir), as shown in **Figure** **1**a. Instead of a conventional recurrent structure, various physical systems that utilize their nonlinear dynamics to work as a computational resource have been discussed extensively.^[^ ^3^, ^4^, ^5^, ^6^, ^7^, ^8^, ^9^, ^10^ ^]^ In fact, any dynamical system has the potential to be a reservoir for information processing.^[^ ^5^ ^]^ This basic concept introduces the concepts of arbitrariness and diversity in the choice of a reservoir and leads to the thriving development of physical reservoir computing.^[^ ^11^, ^12^, ^13^, ^14^, ^15^, ^16^, ^17^, ^18^ ^]^ Hardware implementation of RC utilizes a variety of physical systems, devices and materials, and introduces more possibilities for hardware breakthroughs. Different feats displayed by different systems or designed schemes have been discussed extensively during recent years, among which organic electrochemical devices began to attract an attention. There is a study using semi‐conductive dendritic organic electrochemical networks for bio‐signal classification, proved that the nonlinearities created by electrolyte when electrically excited could be an advantage in RC.^[^ ^19^ ^]^ However, rich dynamic properties of electrochemical reactions have not been widely studied, and their greater application potential deserves to be further explored.

## 2 Design and Realization Scheme

Neuromorphic devices based on molecular dynamics or chemical reactions have great advantages in reducing power consumption and area reduction. The possibility of constructing complex neural networks based on molecular devices has been discussed in our previous research.^[^ ^20^ ^]^ According to this conjecture, we discussed a reservoir construction method that can take advantage of the current‐to‐voltage property presented by molecules in the following research, further demonstrating the ability of chemical dynamics in the field of computing.^[^ ^21^ ^]^ Based on the prior studies, in this paper we build a physical RC system to utilize diverse electrochemical reactions of actual materials and experimentally analyze their computing ability. This section gives the concept of physical RC and the realization scheme of our system.

### 2.1 Concept of Physical RC

From Figure 1a, we can observe that the structure of RC includes input layer, reservoir and output layer. A novel scheme exploring physical dynamics in the reservoir, as a computational resource, was called physical RC, in which the reservoir part is replaced by a physical dynamical system.^[^ ^18^ ^]^ Such a physical dynamical system could act as nonlinear nodes in the traditional reservoir with varying degrees of data processing. Therefore, the physical dynamical system should have sufficiently rich dynamics with high effective dimension to enable diverse responses of the system to the same input. However, to make the reservoir works successfully, there is one prerequisite, that is, the reproducibility of the input–output relation,^[^ ^22^ ^]^ which is also termed an echo state property.^[^ ^23^, ^24^ ^]^ When the system becomes chaotic and exhibits considerable dynamics, it loses the echo state in general and its relevance to the input data, making it an unreliable computing resource. When this condition is satisfied, the output signals behave like a series of signals synchronized with the input series.

The working principle of RC can be described as follows. The output signal $\hat{y}$ should be trained to be as close as possible to the target signal *y* by adjusting the output weights *W* ^out^, and the optimal values can be obtained by a linear regression algorithm, such as the least‐squares method (i.e., minimum $∥y_{\mathrm{train}}-{{\hat{y}}_{\mathrm{train}}}^{2}∥$). Therefore, at the temporal unit *t*, the output signal $\hat{y}\left(t\right)$ and reservoir state *x*(*t*) can be expressed as follows

(1) $$\hat{y}\;\left(t\right)=W^{\mathrm{out}}\cdot x\left(t\right)$$

(2) $$x\left(t\right)=f_{\mathrm{res}}\;\left(W^{\mathrm{in}}\cdot u\left(t\right)+W^{\mathrm{res}}\cdot x\left(t-1\right)\right)$$

where *f*_res_ is the nonlinear function of reservoir, *W* ^in^ and *W* ^res^ are the input weights and weights between nodes, respectively. *u*(*t*) is the input value at the current temporal unit, while *x*(*t* − 1) represents the reservoir state at the previous temporal unit. To quantize the deviation between the target signal and the testing signal as well as to evaluate the performance of RC, mean square error (MSE) is generally used. With the testing data a length of *M*, the expression of MSE is as follows

(3) $$\Delta E=\frac{\sum_{t}{\left(\hat{y}\left(t\right)-y\left(t\right)\right)}^{2}}{M}\;$$

Based on this expression, the normalized mean square error (NMSE) is defined to measure the mean relative scatter and reflect the random errors, expressed as follows

(4) $$\Delta E\;=\frac{1}{M}\;\frac{\sum_{t}{\left(\hat{y}\left(t\right)-y\left(t\right)\right)}^{2}}{\sigma^{2}\left(y\right)}$$

### 2.2 Proposed RC Scheme and Structure Principle

Figure 1b shows the concept of our proposed RC system, which is based on the planar electrodes shown in Figure 1c. The process of obtaining the response data can be simply described as follows: Inject the input voltage signal to one electrode and read the response current signals from the other electrodes as the reservoir state. The solution sample is dropped on the surface of the metal electrodes. Figure 1c shows the testing device, which was composed of 90 pairs of planar electrodes (6 groups × 15 pairs). One side is selected as the input electrodes (e.g., the right) to the input voltage and the other side as the readout electrodes (e.g., the left) to read the response current. One pair of electrodes was recognized as one node in the reservoir. To achieve our design scheme, we introduced a multiway data acquisition system attached to a number of readout electrodes in our testing system to facilitate the control of readout nodes, which also made parallel testing possible. Figure 1d presents a general flowchart: after the input voltage is imposed on the input electrodes, the readout boards (i.e., multiway data acquisition system) consisting of a current‐to‐voltage converter and differential amplifier circuits would transfer the response current to voltage within a range that can be detected. In this case, the response currents from many readout electrodes can be captured using fewer pins. The entire process was controlled by a microcontroller (Arduino Mega2560), and a detailed process description is provided in the Supporting Information. A complex task generally requires a large number of nodes in RC, and the definition of nodes, such as defined by time‐multiplexing and external parameters, greatly affects system performance.^[^ ^13^, ^21^ ^]^ Therefore, selection of appropriate node definition is also one of the most important explorations in physical RC system, and the work in this paper also covers this.

## 3 Working Principle and Verification

**Figure 2:**
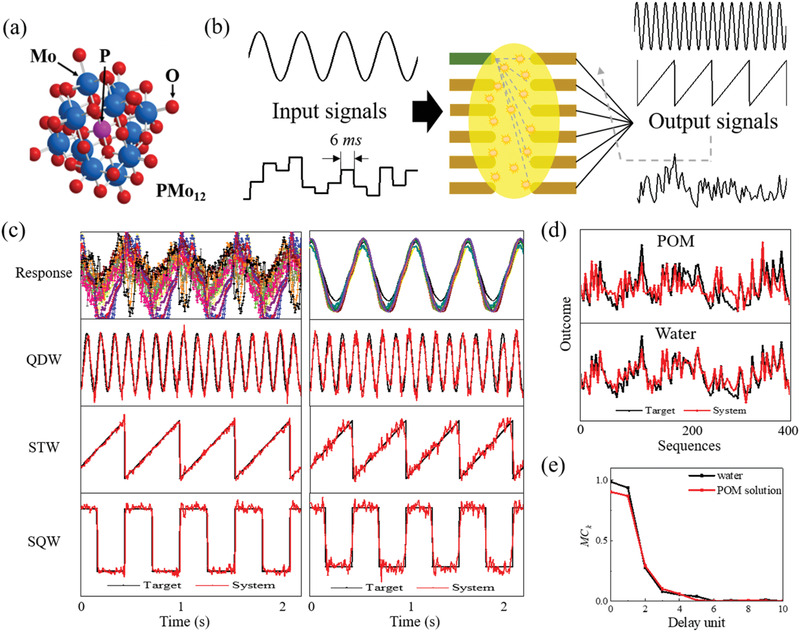
Schematic of electrochemical‐reaction‐based reservoir. a) A structure of the polyoxometalate (POM) molecule. b) Process diagram of two tasks performed by testing systems. c) Responses of POM solution (left) and deionized (DI) water (right) to sinusoidal signal and their predicted performance to target signals of quadruple sine (QDW), saw tooth (STW), and square waves (SQW). d) Predicted performance of POM solution and DI water to a nonlinear target. e) Short term memory for the linear target signal of DI water and POM solution.

Polyoxometalate (POM) exhibits several advantages owing to the unique structure, as shown in **Figure** **2**a. Multiple oxidation and reduction characteristics enrich the dynamics of the RC system as well as promote the information processing capability.^[^ ^20^, ^25^ ^]^ In this study, we utilize the electrochemical current in the solution, of which complex and large nonlinear response is expected even at low voltages owing to the high and multiple redox activities of POMs.^[^ ^26^ ^]^ A simple signal prediction test was performed first to verify the effectiveness of our system and POM molecule, and the general process is shown in Figure 2b. The processing ability of our system is embodied in the prediction results of periodic signals and of a second‐order nonlinear auto‐regressive moving average (NARMA2) model.^[^ ^27^ ^]^ The concentration of the molecule is not specified in this study because the specific concentration values do not result in discernable difference within a certain range. The solution concentrations between 1–3 mg mL^−1^, used for the performance tests, do not affect the results of the experiment; this conclusion is proved succinctly in the last section of Supporting Information.

For periodic signal prediction, the input *u*(*t*) is a sinusoidal signal with a period of 1.8 s, while the target signals are sine wave signals with quadruple frequency of input signal, saw tooth wave signals and square wave signals of the frequency same to input signal, respectively. The response currents were normalized to the range of [0, 1] as the training and testing data, respectively. To evaluate the performance, we calculated the MSEs between the predicted and target values. The response of DI water (Figure 2c, right) to a sinusoidal input signal corresponds to a signal with smooth sinusoidal shape and varying amplitudes, while the POM solution (Figure 2c, left) exhibited more diverse and complicated responses, thus leading to smaller errors. Additionally, with 112 nodes, the MSEs of the POM solution for the three target signals are 0.023, 0.002, and 0.006, and those of DI water are 0.0512, 0.0391, and 0.0565, respectively. These results confirm what we expected, that is, POM can enhance the computing power of our physical system. However, in the NARMA2 task, where the input *u*(*t*) is a random signal with a step width of 6 ms, the prediction performance of the POM solution is inferior to that of DI water. The NMSE was introduced to evaluate the NARMA2 performance, and the calculated results were 0.2106 and 0.3865 for DI water and POM solution, respectively (with 112 nodes). Figure 2d shows the NARMA2 performance of POM solution and DI water.

To analyze the reasons for the inconsistent performance, a short‐term memory capacity (MC) test was performed.^[^ ^28^ ^]^ MC_*k*_ corresponds to the squared correlation coefficients between the current reservoir states (represented as temporal unit, *t*) and *k* ^th^ unit of past input (i.e., (*t – k*), where *k* ∈ [0, 10]), which ranges from 0 to 1. It reflects the extent to which the current states can be explained from past inputs. From this calculation, we found two coefficients in showed significant differences in this task. The results of DI water and POM solution are plotted in Figure 2e. The two coefficients MC_0_ and MC_1_, which play the most important roles in the NARMA2 task, are higher in DI water than in POM solution and yield a better performance in NARMA2 (see Equation S3 in Supporting Information).^[^ ^27^, ^29^ ^]^ Low values of these two coefficients indicate a lack of partial information, which means that the dynamic characteristics caused by redox reaction of POM molecules are too strong and the consistency of information is kind of destroyed. Nevertheless, these results exceeded our expectations because the value of MC_1_ was similar to MC_0_. This was achieved by a “feedforward” parameter in our previous work based on the use of electronic devices which lacked response dynamics, and the values of the parameter was selected based on a series of analyses.^[^ ^21^ ^]^ In this solution RC scheme, this feature existed naturally and was independent of the choice of the solution sample. This highlighted the potential of our system and demonstrated that our system deserves further development. To clarify the differences in these responses as well as further improve the system performance, we examined various measurement parameters.

**Figure 3:**
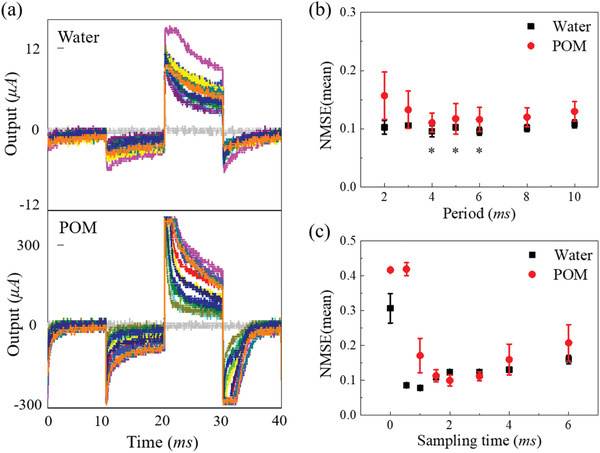
Investigation of computing ability. a) Response signals of DI water and POM solution to a series of input voltages. b) Prediction results of DI water and POM solution at different response periods but with the same sampling time. c) Prediction results of DI water and POM solution for different sampling times at a response period of 6 ms. Plots in b,c) show averaged values using three trials and the error bars show standard deviations.

**Figure** **3**a shows the plotted response waveforms of 15 output electrodes. Data were derived directly from the oscilloscope, wherein we input a series of voltage values to test one specific electrode, and each input voltage was maintained for 10 ms. The current of DI water was significantly smaller than that of the POM solution because of the lack of electrolytes. The measured current through DI water does not exhibit a clear potential window due to the existence of non‐Faradic currents; the electric current flowing in water should be zero within a potential window (i.e., less than ≈1 V) during an ideal electrochemical measurement. An electric double layer induced at the solution interface with gold should contribute to a large capacitive current. Our measurement setup utilized the variation of condition in each pair of electrodes to yield signal response complexity that serves as a computational resource for RC, which was not technically absolute electrochemical information.

The response waveform of the samples shows different transient characteristics. Specifically, the upper and lower voltage limits are due to the limitation of the *I–V* amplifier. The input voltage range and magnitude factor of the amplifier were adjusted to make the output from all nodes the most adequate value on average. Compared with the gradual trend during the entire response of DI water, the response of the POM solution was more intense in the initial response. The abrupt initial spike was caused by the double layer on the electrode surface and by an interaction of the capacitive resistance circuits within the parallel external circuit, as explained in the first section of Supporting Information. It is considered that the intense capacitive current led to a poor POM solution computing power at early sampling times.

From the observation of the response waveform, we speculated that the performance of this system may be affected by the length of the reaction time, so it was necessary to further control the parameters of the time. This is also the exploration of the more appropriate node definition that mentioned earlier. To clarify how the reaction time of the solution affects the computing power, we tested the performance of the NARMA2 task in terms of the test period and sampling time, and the statistical results are plotted in Figure 3b,c, respectively. In Figure 3b, the sampling time was kept at 1 ms for DI water and at 2 ms for the POM solution. In Figure 3c, the response period was maintained at 6 ms in both samples. In addition, the results of the two samples in Figure 3b were obtained from the same set of electrodes, and Figure 3c was from another set of electrodes to ensure the consistency of the test conditions. Both sets of electrodes were brand new, and DI water was tested first before POM solution because the redox reaction of the POM solution would cause damage to the electrode surface thus affects the stability and reliability of the results. At the end of all tests, there was no serious damage to the electrode surface. However, after numerous measurement repetitions, we found that the response current of the solution started to increase significantly. To avoid the interference attributed to this factor, we changed the testing solution for each test from the same prepared solution sample. Moreover, in Figure 3b the three tests for two samples (24 NMSEs in total) at sampling period of 3 to 6 ms are marked with an asterisk to indicate the data used for analyzing variance. The calculated P‐value of 0.310199 indicates the response of the two samples to be no significant difference under their respective optimum settings in NARMA2. Figure 3b indicates that the response period had no correlation with the computing power of DI water, and only affected that of the POM solution when the period was less than 4 ms. Figure 3c reveals the dependence of computing power on sampling time for both samples: the best sampling time (the lowest error and most stable performance) for DI water was around 1 ms, and for POM solutions was ≈2 ms. Although the lower optimal sampling time for DI water than that for POM can be attributed to the rapid migration of protons and hydroxide ions during the conduction of electricity, it is difficult to investigate the details clearly at this stage because their migration process remains largely unknown.^[^ ^30^ ^]^ There is no doubt, however, that proton migration plays a positive role in this process, as the system computing power deteriorates in the case of non‐protonic solvent dimethylformamide (see the first section of Supporting Information).

**Figure 4:**
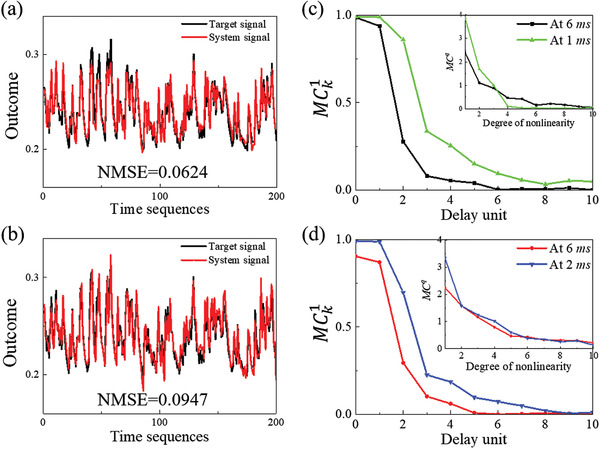
Comparison of DI water and POM solution outcomes. Prediction performance and prediction errors of a) DI water and b) POM solution. Short‐term memory to first‐order target and the memory capacity (inset) of c) DI water, d) POM solution.

From the above analysis, we fixed the response period to 6 ms and repeated the same tests many times. Finally, we got the conclusion that better computational performance can be achieved by selecting better reading time points. For example, in NARMA2 task, NMSE of DI water was decreased from 0.2106 to 0.0624, and that of POM solution greatly decreased from 0.3865 to 0.0947. **Figure** **4**a,b shows the best NARMA2 results of DI water and POM solutions, respectively. Their corresponding MC calculation results are plotted in Figure 4c,d, which incorporated the MC results of Figure 2e for comparison purposes. In addition to the linear MC calculation, we use the Legendre polynomial to construct the target signal *P_q_* (*t* − *k*) in order to calculate the MC of higher order nonlinearities, where *q* is the degree of polynomial. The sum of MC_*k*_ values at the same *q* of past units (*t – k*) is labeled as MC^*q*^.^[^ ^5^ ^]^ This expression was used to extend the MC measurement for evaluating the nonlinear memory capacities, and the results are shown in the insets of Figure 4c,d. The results confirm that MC^1^ (i.e., the linear MC results), especially the ${\mathrm{MC}}_{1}^{1}$ and ${\mathrm{MC}}_{2}^{1}$ (i.e., the preceding MC_0_ and MC_1_), could be greatly increased by adjusting the sampling time. It did not affect the MC*^q^* of the POM solution for higher‐order signals (i.e., *q* ≥ 2), but it had an adverse effect on the higher‐order MC*^q^* of DI water.

The information processing capability of our system was evaluated using a real‐world task which was pneumatic artificial muscle (PAM) length prediction.^[^ ^31^ ^]^ PAM is well known to exhibit hysteresis, and to estimate its length, we need to consider the history of applied pressures. Therefore, this task is appropriate to evaluate the memory‐required computational capability. In order to perform the investigation, 3000 data points were chosen from the open‐source data file provided by Akashi et al.^[^ ^29^ ^]^ The training and testing processes were maintained the same to the NARMA2 task, and NMSE was used to represent the error. The results from Figure 3c were used to select the readout time; the magnitudes of current in DI water were measured at 1 ms intervals, and that in POM solution were measured at 2 ms intervals. The duration of each input voltage was 4 ms, and 112 nodes were used, consistent with the previous tests. The NMSE values were obtained as 0.1102 from DI water and 0.2507 from POM solution, which are consistent with the NARMA2 results and MC calculations. A previous work reported the NMSE values, from the echo state network, between 0.28 to 0.003 using only air pressure to emulate the PAM length. Although the values obtained in this work are slightly lower, these results were obtained using a natural physical computing system rather than a software‐based neural network. Furthermore, we only used 112 nodes for the computations compared to their 3000. Additionally, both results were significantly better than those predicted by linear regression system, which indicates that our system is capable of implementing the nonlinearity and memory capacity required for this task. The present work also demonstrates the strong computing power of distilled water. Therefore, with further development of our system, DI water can be competent for this task. The procedure of analysis are discussed in the first section of the Supporting Information and the results are shown in and Figure S1c, Supporting Information.

**Figure 5:**
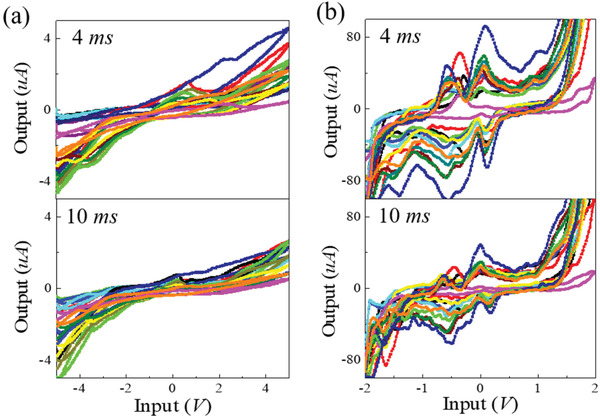
*I–V* characteristics at two testing periods. The sampling point was at 4 ms (upper graphs) and 10 ms (lower graphs) of a) DI water, b) POM solution.

To explore the effect of reaction time on the current‐to‐voltage response, we tested the *I–V* characteristics of these samples at two periods equal to 4 and 10 ms, respectively. The current curves plotted in **Figure** **5** are similar in shape to the cyclic voltammetry curves.^[^ ^32^ ^]^ However, the shapes are not exactly the same with respect to the peak position and magnitude ratio. The two‐terminal measurement lost definitive control of the potential at the working electrode surface; therefore, the chemical reaction in our system took place at input voltages which deviated slightly from the norm at each pair of electrodes with different distances. The complex but reproducible higher nonlinearity of the current from the POM solution was advantageous in the periodic signal reconstruction task. Conversely, the *I–V* characteristics of DI water exhibit less nonlinearity but greater help to higher order tasks. This is directly reflected in NARMA2 task and PAM length task: the dynamics offered by chemical reactions of POM were less helpful to performance than proton migration in DI water.

Our system provides a novel perspective on the reservoir application of Faradic currents in solution. Although an organic‐electrochemical‐reservoir has recently been reported,^[^ ^19^ ^]^ the detected signal is the electrical current flowing through an organic‐ionic‐electronic conductor, where the organic fibers within the electrolyte create strong nonlinear responses corresponding to multiple input signals. Our system detects the formation and transients of the electric double layer along with the electrochemical reaction. This results in good computing performance even at fast measurement speeds, and the power consumption is also expected to reduce owing to the dynamics of ions. Considering the high sensitivity of the computing power of electrochemical reaction to various factors, a more rigorous exploration ought to be conducted to maximize its computing ability, and to enhance the coupling of the functionality of complex chemical reactions and the dynamics of light ions.

## 4 Conclusion

With the help of a multiway data acquisition system and electronic circuits, we realized a physical RC system based on ionic current or proton migration. This system displayed “feedforward connection” between nodes, which was proved to be independent of sample selection in this study. However, in our previous scheme, which was designed according to the nonlinear *I–V* characteristics of molecules, this condition was achieved by introducing external parameters of “feedforward gain.”^[^ ^21^ ^]^ We think this is a natural advantage of our system and deserves further development and exploitation. Moreover, we found that the complex and diverse chemical reactions of POM molecules contributed to the enhancement of information processing ability for periodic signals and memory in higher‐order, nonlinear systems, but the computing power of higher‐order tasks has not been fully utilized. Conversely, DI water showed strong computing power in the NARMA2 task. Other studies have shown that good performance in time‐serials prediction task also enables handwriting font recognition, isolated word recognition, and other classification tasks.^[^ ^13^, ^14^, ^19^, ^21^ ^]^ Therefore, our proposed device has the potential to serve as a computing system. Furthermore, in this device with the solution sample, electrons barely flow from the electrodes to the surface of the material owing to the fact that protons travel much faster than ions.^[^ ^30^ ^]^ Based on this discovery, it may be possible to design a more powerful computing system using the proton or ion transfer with minimal electrochemical reactions within very fast time sequential range to provide a low‐cost, low‐power consumption, and highly integrated hardware devices for increasingly important edge computing. We hope that our research provides a new perspective combining chemical properties for the future development of hardware computing systems with low‐power consumption.

## Conflict of Interest

The authors declare no conflict of interest.

## Supporting information

Supporting Information

## Data Availability

The data that support the findings of this study are available from the corresponding author upon reasonable request.
