# Supplementary material for: Physical Implementation of Reservoir Computing through Electrochemical Reaction
Source: Adv Sci (Weinh). 2021 Dec 29;9(6):2104076. doi: 10.1002/advs.202104076 (PMC8867144; doi:10.1002/advs.202104076)
Supplement: Supplementary file 1 — Supporting Information [file ADVS-9-2104076-s001.pdf]

WILEY-VCH

ADVANCED SCIENCE
Open Access

Supporting Information

for *Adv. Sci.*, DOI: 10.1002/advs.202104076

Physical Implementation of Reservoir Computing through Electrochemical Reaction

*Shaohua Kan, Kohei Nakajima, Tetsuya Asai, and Megumi Akai-Kasaya**

Supplementary Material:

Physical Implementation of Reservoir Computing through Electrochemical Reaction

*Shaohua Kan, Kohei Nakajima, Tetsuya Asai, and Megumi Akai-Kasaya**

Herein, we supplement the main text listings, including the definition of performance evaluation indicators, training and testing methods, the setting of specific parameters, and others.

I. Supplement to the performance and testing results

We declared in the text that the prediction ability of the Polyoxometalate (POM) solution to periodic signals improved (Figure 2(b)) compared with that of distilled (DI) water. In this task, the input sinusoidal signal had 100 periods in total, and each included 300 time steps. Each time step lasted for 6 *ms*. The response currents were normalized in the range of [0, 1] for training and testing, as described in the next section.

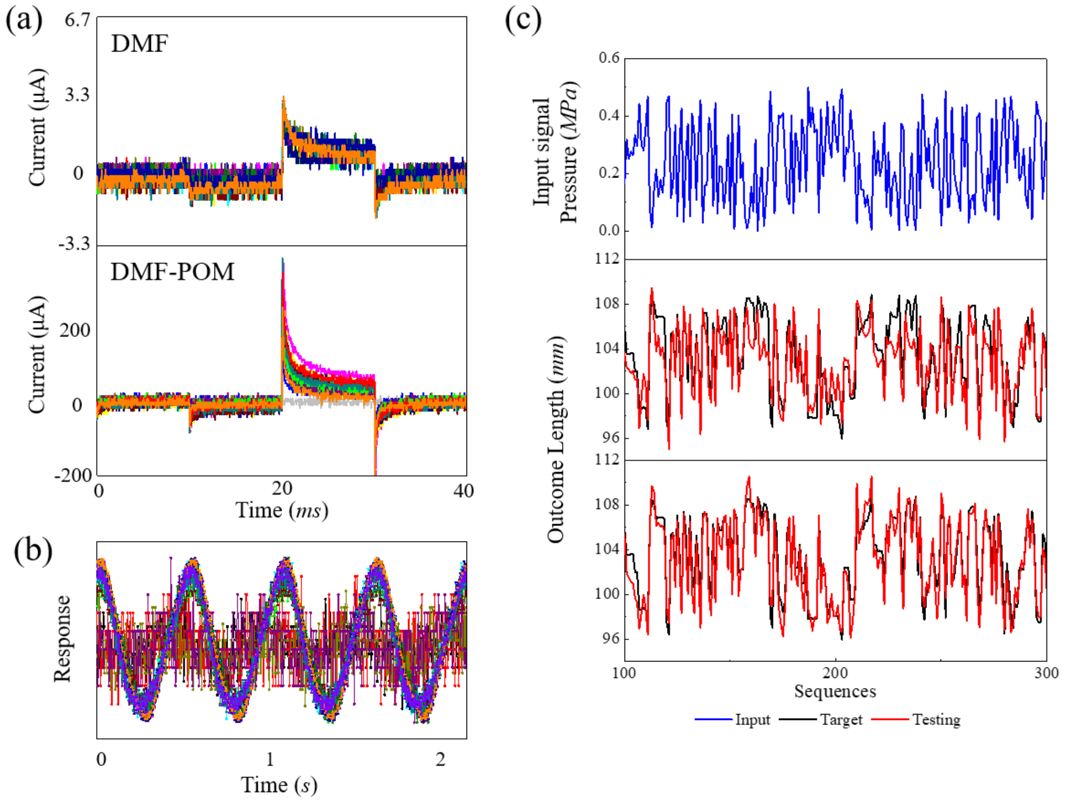

Figure S1 (a) Current responses of dimethylformamide (DMF) and DMF with Polyoxometalate (POM). (b) Responses of DI water to sinusoidal signal in which four readout pins are in the "OFF" state. (c) Presentation of pneumatic artificial muscle (PAM) length task: input pressure (top) as well as results of POM solution (middle) and DI water (bottom).

Figure 3(a) shows the current responses of DI water and POM solution with the original data captured by the oscilloscope. In the main text we declared that proton migration should play a significant role in the information processing capacity of the system. For comparison, we chose the nonprotonic solvent dimethylformamide (DMF) to test its current response waveform with and without POM, as shown in **Figure S1(a)**. This was under the same process that led to Figure 3(a): imposed a string of input voltages (i.e., five random numbers) on one input electrode and read the current responses of 15 output electrodes. Similar to the effect of POM in DI water, the addition of POM in DMF could greatly increase the current response and cause an initial spike in the response. However, the addition of POM did not make the responses at each electrode more distinct as it did in DI water, and it did not show any advantage in computing ability of DMF and DMF with POM. Thus, we believe that proton transfer is worthy of further study in the future.

Not all parts of the output current shown in these four figures are the response currents from the solutions. For example, the light gray output in the four graphs in Figure 3(a) and Figure S1(a) is an invalid node with no readout value. This was owing to an improper working state of the device, that is, the selector circuit used in the readout boards did not switch the specific port. Such an invalid node is acceptable in RC, so we kept these invalid nodes in the NARMA tasks and MC calculation. However, we eliminated this part of the response current from the sinusoidal periodic prediction task. This is because a weak current can still be read out even if the port is not "ON" because of the existence of leakage current on the circuit, and the weak current will lead in phase compared with the current that is being normally read out (i.e., when the switch is "ON"). We illustrate the responses of DI water in both cases in **Figure S1(b)**, whereby the four weak, noisy, and phase-shifting waveforms are the read leakage currents. It is obvious that this feature is detrimental to the processing of periodic signals. Furthermore, we experimentally investigated the abrupt initial spikes shown in the insets of Figure 3(a) and Figure S1(a) on the breadboard. By connecting a series of side-by-side resistors on the breadboard with a series of readout ports on one output board, we compared the output current with and without the neighboring resistors. The results show that the spikes appeared on the readout port when a resistor was also inserted on the neighbor port, and became larger with an increasing number of neighbors with a resistor. Therefore, in the system with solution, POM caused capacitive resistance circuits on the parallel electrodes of our device, which interacted with the adjacent electrodes, and resulted in an initial current spike.

The behavior of pneumatic artificial muscle (PAM) can hardly control because of their mechanical nonlinearity and hysteresis. However, their intrinsic dynamics could be learned well by reservoir computing. Therefore, we introduced this task in our work to further evaluate the computing ability of our system in a real task. The data we used was from an article entitled as "Input-driven bifurcations and information processing capacity in spintronics reservoirs". In this task, the input data, air pressure (the blue data in the upper **Figure S1(c)**), will be transferred into the voltage from -2 to 2 *V* input into our system. The period of each input voltage is 4 *ms*, and read the current of DI water at 1 *ms*, and read that of POM solution at 2 *ms*. The number of nodes, length of training data and testing data, and other parameters are consistent with those in NARMA2 task. POM solution and distilled water test results are also shown in Figure S1(c): the middle illustration shows the prediction result of POM solution and the bottom one shows that of DI water. Their NMSEs are 0.2507 and 0.1102 respectively.

II. Training and testing process

Two training methods were used in this study. One was the least-squares method used for the training of periodic target signals, and the other was ridge regression, which was used for the training of high-order target signals. An input signal with M data (M = 3000 in this study) was applied to a reservoir with N (N = 112 in this study) nodes produced $M \times N$ reservoir states X. In this case, an output signal $\hat{y}$ with M data can be obtained by multiplying these reservoir states by N output weights W_{out}. The training aim was the minimization of the objective function $\|y_{train} - \hat{y}_{train}\|^2$, where subscript "train" refers to the training part of the target signal and output signal.

For the least square method, the output weight W_{out} was calculated by,

$$W_{out} = ({X_{train}}^T X_{train})^{-1} {X_{train}}^T y_{train} \quad \text{(S1)}$$

The other method used for the NARMA task and MC calculation was ridge regression. Compared with the least-squares method, it artificially adds a non-negative factor k to the main diagonal element of the independent variable matrix, as follows,

$$W_{out} = ({X_{train}}^T X_{train} + kI)^{-1} {X_{train}}^T y_{train} \quad \text{(S2)}$$

The predicted output signal is obtained by multiplying the testing signal by the output weights calculated from the training signal. The prediction performance is evaluated by comparing the deviation between the predicted and target signals. In the training process of periodic signal prediction, the first 20 data points were used as the initial states to initialize the reservoir, 2500 as the training part, and 400 as the testing part. The remaining 80 data points

were ignored because several predicted values of them sometimes were far off the target. In the NARMA task, the first 100 data points were used for the initialization of the reservoir, 2500 for the training part, and the remaining 400 for the testing part.

Ridge regression used a non-negative factor k, and different values of k were used for different situations. k was set to the value of 0.1 for the NARMA2 task, and to the value of 1 for the MC task.

III. Definitions of performance evaluation

1. Definition of NARMA2 model

The NARMA task is a higher-order dynamical model constructed from the current and previous inputs, and was extensively used to evaluate the reservoir's ability to duplicate the NARMA signal. The number following the letter "NARMA" represents the order of the model. The expression for NARMA2 model is defined as follows.

$$y(t+1) = 0.4y(t) + 0.4y(t) \cdot y(t-1) + 0.6u(t)^3 + 0.1 \quad \text{(S3)}$$

where *y* in the expression is the target signal following the model, (t - k) represents the state which is k units ahead of the current time t (t is dimensionless and represents the sequence number), and u is the input signal with 3000 random sequences in the interval of [0, 0.5].

2. Definition of memory capacity

Memory capacity (MC) calculates the squared correlation coefficient which quantifies the correlation between the current reservoir states (at time t) and past inputs (at time (t - k)), ranging from 0 to 1. In the MC task, the input signal u is the same random number sequences used in the NARMA task, but mapped from its original range [0, 0.5] to [-1, 1]. The target signal y_k is constructed from the kth delayed input signal (i.e., $y_k = u(t-k)$). The training and testing method is ridge regression, as explained in the previous section. The kth-delay MC_k is expressed as

$$MC_k = \frac{cov^2(y_k, \widehat{y_k})}{\sigma^2(y_k) \cdot \sigma^2(\widehat{y_k})} \quad \text{(S4)}$$

Specifically, we measured MC_ks with delayed units from 0 to 10 for a higher-order target signal following the Legendre polynomials, labeled as MC_k^q. The superscript q represents the degree of nonlinearity of the target signal y_k to the kth delayed input signal $u(t - k)$. Short-term memory capacity is the sum over all MC_k with the same q, and is denoted as MC^q

$$MC^q = \sum_{k=1}^{\infty} MC_k^q \quad \text{(S5)}$$

III. Detailed description of testing processing

In this section, we provide a detailed explanation of the process flow shown in Figure 1(d). Arduino Mega 2560, as the main controller in our testing system, takes charge of sending the input, control, and receiving response signals. Arduino sent the “begin transmission” order and the input voltage (digit value) to a digital-to-analog converter (DAC) PCF8591, and the analog voltage can then be obtained from the DAC. Because the output range from the DAC is in the range of 0–4 *V*, we also used a differential amplifier circuit to convert the voltage to -2 to 2 *V* and then amplify it; the amplification factor can be adjusted freely between 1 and 3. In this case, voltages from negative to positive values are imposed on the input electrodes. Following the imposed voltage, the response current was detected on the readout electrodes. Each readout electrode was connected to a corresponding pin of the readout boards, with a total of 48 pins (six readout boards, each with eight pins). The integrated current to voltage converter and differential amplifier circuit on each readout board converted the response current read to a voltage of 0 to 4 *V* owing of the reading range limitation of Arduino analog ports. There are three options for the magnification factor: 10^4, 10^5, and 10^6. The converted voltage on eight ports of each board will be selected in turn through our 8-way selector circuit, which includes eight switches controlled by the shift register SN74HC595. The Arduino sends the control signal to the shift register to alternately select one switch out of eight to turn on to transfer the conversion voltage of the corresponding port to the output port. In this case, the response currents from a total of 48 readout electrodes on the six boards can be captured through just six analog pins on the Arduino.

IV. Composition of the test end of the electrode

Figure S2 shows an array of electrodes. One testing board was composed of 90 pairs of rounded-tip electrodes, each with a gap of 50 μm. The distance between adjacent electrodes was 400 μm. Each electrode was 80 μm wide, and their corresponding terminals were wire-bonded to external hard wirings. The electrodes, composed of Cr/Pd/Au, were deposited on Pyrex glass and then covered by a polyimide film that left the facing area bare.

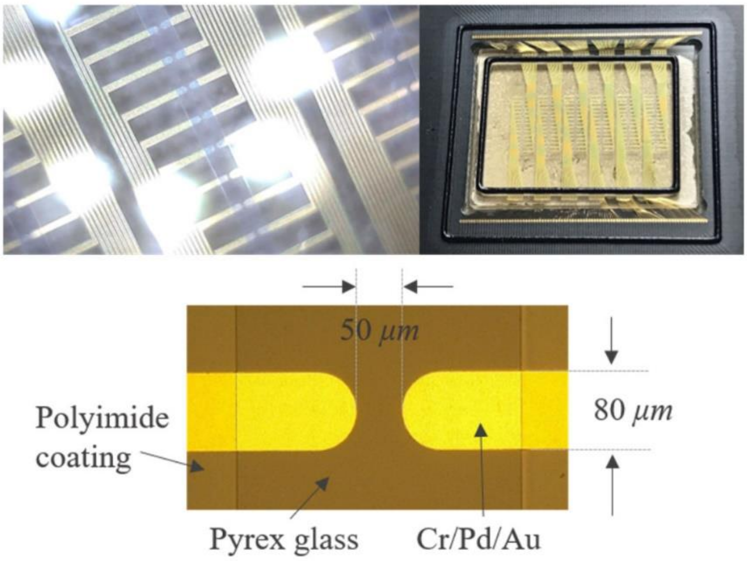

Figure S2 Actual electrode array graph and the magnified parts of one of its pairs. The test end of electrode array for polymer wire growth consists of 90 pairs of rounded-tip Au electrodes with a gap of 50 μm. The wiring area is covered by polyimide film.

V. Instructions on selection of POM solution concentration

In the main text, we stated that the concentration of POM solution was not specified strictly in this study. However, this does not mean that the selection of solution concentration is arbitrary or unreliable. During our testing, we found that there was no significant difference in current responses for solution concentrations greater than 1 *mg/mL* (but not greater than 10 *mg/mL*). In this section, we did a simple proof. The cyclic current responses at different solution concentrations was tested on a new set of electrodes. The cycle voltage at each concentration was repeated 100 times, and the cycle data of the last time was selected. This allows the electrochemical reaction to go on for a while, so that conform to our performance testing process shown in the text. Eight groups of different solution concentrations were selected for testing. After each test, the surface of the electrodes was washed with distilled water and sucked it dry before the new test solution was added. The results of this test are shown in **Figure S3**.

The middle illustration in Figure S3 is the statistical results. The black dots represent the difference between the maximum and minimum value of each curve, while the red dots are the peak difference between the two most prominent REDOX peaks during voltage increase and decrease. The 8 illustrations surrounding the middle one are the circulating currents at 8 sets of solution concentrations, which are 0, 0.5, 1, 2, 3, 4, 6, 8 *mg/mL*, respectively. The voltage increases and decreases at 0.2 *V* intervals between -2 and 2 *V*, each voltage value

lasting 2 *ms* then readout the current. Intuitively, there is no significant difference between the cyclic voltammetry curves between 1 and 8 *mg*/*mL*. This may be due to the fact that only about 0.1 *mL* of the solution is dropt on the electrode surface, making the difference in the

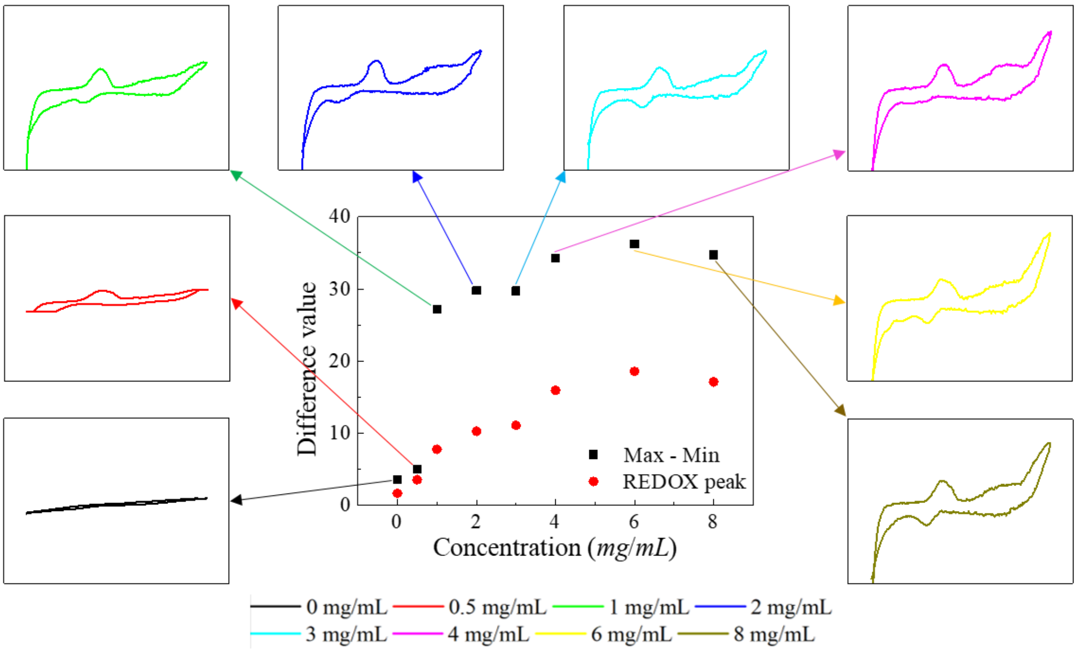

Figure S1 I-V characteristics of POM solution at different concentrations from a fixed pair of electrodes. 8 surrounding illustrations have the same axis values: x-axis is the input voltages from -2.5 to 2.5 *V*, y-axis is the output currents from -20 to 25 μA.

amout of solute insignificant.
